# Supplementary material for: Time-series transcriptome analysis identified differentially expressed genes in broiler chicken infected with mixed Eimeria species
Source: Front Genet. 2022 Aug 8;13:886781. doi: 10.3389/fgene.2022.886781 (PMC9393255; doi:10.3389/fgene.2022.886781)
Supplement: Supplementary file 2 [file DataSheet1.ZIP › 7dpi_GO.Gsea.1625106495923/GOBP_ATP_SYNTHESIS_COUPLED_ELECTRON_TRANSPORT.html]

Details for gene set GOBP\_ATP\_SYNTHESIS\_COUPLED\_ELECTRON\_TRANSPORT[GSEA]

|  || Dataset | TMM\_7dpi\_gct\_format\_7dpi\_gct\_format.Class\_4dpi.cls #PC\_versus\_NC.Class\_4dpi.cls #PC\_versus\_NC\_repos |
| Phenotype | Class\_4dpi.cls#PC\_versus\_NC\_repos |
| Upregulated in class | 1 |
| GeneSet | GOBP\_ATP\_SYNTHESIS\_COUPLED\_ELECTRON\_TRANSPORT |
| Enrichment Score (ES) | 0.7766217 |
| Normalized Enrichment Score (NES) | 3.6632462 |
| Nominal p-value | 0.0 |
| FDR q-value | 0.0 |
| FWER p-Value | 0.0 |
Table: GSEA Results Summary

  

Fig 1: Enrichment plot: GOBP\_ATP\_SYNTHESIS\_COUPLED\_ELECTRON\_TRANSPORT      
 Profile of the Running ES Score & Positions of GeneSet Members on the Rank Ordered List

  

| SYMBOL | TITLE | RANK IN GENE LIST | RANK METRIC SCORE | RUNNING ES | CORE ENRICHMENT || 1 | DGUOK | na | 238 | 1.001 | 0.0088 | Yes |
| 2 | COX5A | na | 300 | 0.942 | 0.0307 | Yes |
| 3 | NDUFA2 | na | 303 | 0.935 | 0.0574 | Yes |
| 4 | NDUFB6 | na | 307 | 0.933 | 0.0839 | Yes |
| 5 | NDUFS5 | na | 308 | 0.932 | 0.1106 | Yes |
| 6 | NDUFB7 | na | 323 | 0.915 | 0.1357 | Yes |
| 7 | NDUFB2 | na | 348 | 0.885 | 0.1591 | Yes |
| 8 | NDUFAB1 | na | 356 | 0.876 | 0.1836 | Yes |
| 9 | NDUFA7 | na | 371 | 0.857 | 0.2070 | Yes |
| 10 | NDUFC2 | na | 394 | 0.834 | 0.2291 | Yes |
| 11 | UQCR10 | na | 444 | 0.785 | 0.2475 | Yes |
| 12 | CYCS | na | 475 | 0.763 | 0.2669 | Yes |
| 13 | COX6A1 | na | 480 | 0.760 | 0.2884 | Yes |
| 14 | UQCR11 | na | 485 | 0.754 | 0.3097 | Yes |
| 15 | UQCRB | na | 487 | 0.753 | 0.3312 | Yes |
| 16 | NDUFA11 | na | 497 | 0.746 | 0.3519 | Yes |
| 17 | NDUFA13 | na | 557 | 0.708 | 0.3672 | Yes |
| 18 | NDUFS3 | na | 623 | 0.675 | 0.3811 | Yes |
| 19 | NDUFB3 | na | 656 | 0.657 | 0.3973 | Yes |
| 20 | NDUFS8 | na | 661 | 0.653 | 0.4157 | Yes |
| 21 | NDUFS6 | na | 686 | 0.642 | 0.4321 | Yes |
| 22 | SDHD | na | 708 | 0.632 | 0.4485 | Yes |
| 23 | UQCC3 | na | 735 | 0.620 | 0.4641 | Yes |
| 24 | NDUFA1 | na | 742 | 0.616 | 0.4813 | Yes |
| 25 | UQCRHL | na | 788 | 0.594 | 0.4945 | Yes |
| 26 | NDUFA5 | na | 794 | 0.593 | 0.5111 | Yes |
| 27 | GHITM | na | 842 | 0.575 | 0.5237 | Yes |
| 28 | DNAJC15 | na | 863 | 0.566 | 0.5383 | Yes |
| 29 | NDUFB5 | na | 902 | 0.554 | 0.5510 | Yes |
| 30 | SDHAF2 | na | 919 | 0.550 | 0.5654 | Yes |
| 31 | COX6C | na | 930 | 0.546 | 0.5803 | Yes |
| 32 | COQ9 | na | 939 | 0.541 | 0.5951 | Yes |
| 33 | NDUFV3 | na | 988 | 0.527 | 0.6062 | Yes |
| 34 | NDUFB4 | na | 1015 | 0.517 | 0.6188 | Yes |
| 35 | NDUFA12 | na | 1023 | 0.513 | 0.6330 | Yes |
| 36 | COX7B | na | 1055 | 0.501 | 0.6448 | Yes |
| 37 | NDUFA6 | na | 1079 | 0.492 | 0.6570 | Yes |
| 38 | NDUFA8 | na | 1080 | 0.492 | 0.6711 | Yes |
| 39 | UQCRQ | na | 1103 | 0.484 | 0.6831 | Yes |
| 40 | NDUFS2 | na | 1124 | 0.477 | 0.6951 | Yes |
| 41 | COX4I1 | na | 1125 | 0.476 | 0.7088 | Yes |
| 42 | SDHC | na | 1135 | 0.473 | 0.7216 | Yes |
| 43 | NDUFB10 | na | 1215 | 0.450 | 0.7279 | Yes |
| 44 | UQCRFS1 | na | 1263 | 0.437 | 0.7365 | Yes |
| 45 | NDUFV1 | na | 1307 | 0.428 | 0.7452 | Yes |
| 46 | NDUFB8 | na | 1330 | 0.423 | 0.7555 | Yes |
| 47 | NDUFS7 | na | 1371 | 0.412 | 0.7640 | Yes |
| 48 | NDUFV2 | na | 1395 | 0.408 | 0.7737 | Yes |
| 49 | BID | na | 1694 | 0.334 | 0.7584 | Yes |
| 50 | UQCRC2 | na | 1700 | 0.332 | 0.7675 | Yes |
| 51 | COX7A2L | na | 1761 | 0.316 | 0.7715 | Yes |
| 52 | NDUFB9 | na | 1842 | 0.299 | 0.7734 | Yes |
| 53 | COX15 | na | 1927 | 0.282 | 0.7744 | Yes |
| 54 | NDUFB1 | na | 1995 | 0.272 | 0.7766 | Yes |
| 55 | TAZ | na | 2298 | 0.223 | 0.7577 | No |
| 56 | UQCRC1 | na | 2326 | 0.217 | 0.7617 | No |
| 57 | NDUFA4 | na | 2726 | 0.160 | 0.7329 | No |
| 58 | NDUFA9 | na | 2879 | 0.141 | 0.7242 | No |
| 59 | ISCU | na | 2900 | 0.138 | 0.7265 | No |
| 60 | COX10 | na | 3408 | 0.073 | 0.6861 | No |
| 61 | NDUFA10 | na | 3412 | 0.073 | 0.6880 | No |
| 62 | SDHA | na | 3488 | 0.064 | 0.6835 | No |
| 63 | NDUFS1 | na | 3763 | 0.033 | 0.6615 | No |
| 64 | PARK7 | na | 3768 | 0.032 | 0.6621 | No |
| 65 | NDUFAF1 | na | 3921 | 0.014 | 0.6498 | No |
| 66 | CDK1 | na | 4341 | -0.035 | 0.6157 | No |
| 67 | NDUFS4 | na | 4402 | -0.040 | 0.6118 | No |
| 68 | COX7C | na | 4881 | -0.090 | 0.5744 | No |
| 69 | SNCA | na | 5117 | -0.114 | 0.5580 | No |
| 70 | DLD | na | 5596 | -0.158 | 0.5225 | No |
| 71 | CCNB1 | na | 8681 | -0.511 | 0.2788 | No |
Table: GSEA details [plain text format]

  

Fig 2: GOBP\_ATP\_SYNTHESIS\_COUPLED\_ELECTRON\_TRANSPORT      
 Blue-Pink O' Gram in the Space of the Analyzed GeneSet

  

Fig 3: GOBP\_ATP\_SYNTHESIS\_COUPLED\_ELECTRON\_TRANSPORT: Random ES distribution      
 Gene set null distribution of ES for **GOBP\_ATP\_SYNTHESIS\_COUPLED\_ELECTRON\_TRANSPORT**
